# Group 2 Innate Lymphoid Cells in Respiratory Allergic Inflammation

**DOI:** 10.3389/fimmu.2019.00930

**Published:** 2019-06-07

**Authors:** Sofia Helfrich, Barbara C. Mindt, Jörg H. Fritz, Claudia U. Duerr

**Affiliations:** ^1^Institute of Microbiology, Infectious Diseases and Immunology, Charité – Universitätsmedizin Berlin, Berlin, Germany; ^2^Department of Microbiology & Immunology, McGill University, Montréal, QC, Canada; ^3^McGill University Research Centre on Complex Traits (MRCCT), McGill University, Montréal, QC, Canada; ^4^FOCiS Centre of Excellence in Translational Immunology (CETI), McGill University, Montréal, QC, Canada; ^5^Department of Physiology, McGill University, Montréal, QC, Canada

**Keywords:** group 2 innate lymphoid cells (ILC2s), mucosal immunity, respiratory tract, allergic inflammation, therapeutic strategies

## Abstract

Millions of people worldwide are suffering from allergic inflammatory airway disorders. These conditions are regarded as a consequence of multiple imbalanced immune events resulting in an inadequate response with the exact underlying mechanisms still being a subject of ongoing research. Several cell populations have been proposed to be involved but it is becoming increasingly evident that group 2 innate lymphoid cells (ILC2s) play a key role in the initiation and orchestration of respiratory allergic inflammation. ILC2s are important mediators of inflammation but also tissue remodeling by secreting large amounts of signature cytokines within a short time period. Thereby, ILC2s instruct innate but also adaptive immune responses. Here, we will discuss the recent literature on allergic inflammation of the respiratory tract with a focus on ILC2 biology. Furthermore, we will highlight different therapeutic strategies to treat pulmonary allergic inflammation and their potential influence on ILC2 function as well as discuss the perspective of using human ILC2s for diagnostic purposes.

## Introduction

**Figure 1:**
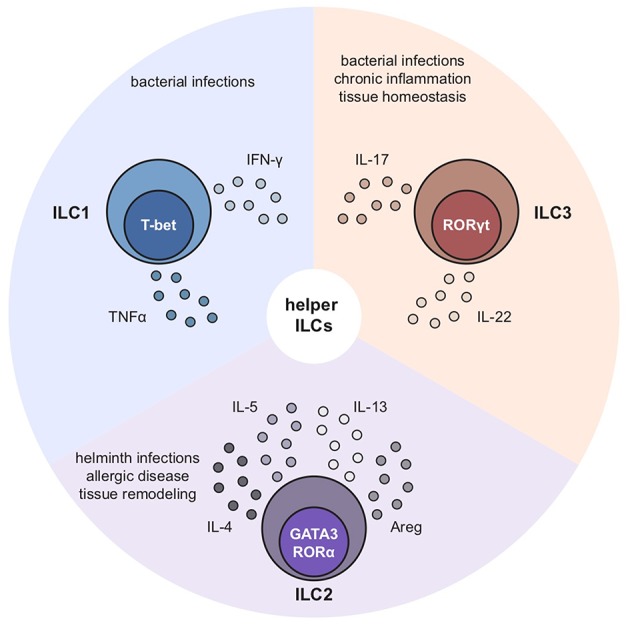
Characteristics of ILC1s, ILC2s, and ILC3s. ILC1s, ILC2s, and ILC3s are characterized by expression of their key transcription factors and signature cytokines: ILC1s depend on T-bet and produce IFNγ, ILC2s on Gata3 and Rorα and secrete IL-4, IL-5, IL-13 as well as amphiregulin while ILC3s depend on Rorγt and release IL-17 and IL-22 upon activation.

Respiratory allergic inflammatory conditions such as asthma and allergic rhinitis (hay fever) are affecting millions of people globally (1, 2). Importantly, the prevalence of these non-communicable disorders is rapidly increasing in contrast to communicable diseases of the respiratory tract, which are on the decline (1). Due to their widespread morbidity, they represent a substantial social as well as economic burden (3). The skew toward a type 2 immune response is often an important characteristic of these chronic conditions that involve both innate and adaptive branches of the immune system (4). In addition to T helper 2 (T_H_2) cells of the adaptive immune system, group 2 innate lymphoid cells (ILC2s) are critical in instructing a strong type 2 immune response (5). ILC2s belong to the group of innate lymphoid cells (ILCs) that provide host defense against infectious agents, participate in inflammatory responses and mediate lymphoid organogenesis and tissue repair, particularly at mucosal barriers. Taking the newest nomenclature of ILCs into account, the ILC family is comprised of five subsets including Natural Killer (NK) cells, lymphoid tissue inducer cells (LTi cells) as well as the three helper ILC members. While LTi cells are key drivers of lymphoid organogenesis and NK cells are important to fight off viral infections, helper ILCs are regarded as the innate counterpart of T_H_ cells but lack the surface expression of common adaptive lineage markers as well as specific antigen receptors (6, 7). These helper ILCs, namely, ILC1s, ILC2s, and ILC3s are defined by their effector cytokine profile and transcription factor expression (8). The master transcription factors for the different helper subsets are T-bet for ILC1, Gata3 and Rorα for ILC2s and RORγt for ILC3s. Helper ILCs are important sources of innate effector cytokines such as ILC1-derived IFNγ, ILC2-derived IL-4, IL-5, IL-13, and amphiregulin (Areg) as well as ILC3-derived IL-17 and IL-22 (Figure 1). ILC2s are innately committed to type 2 immunity which consequently puts them in the spotlight during the onset of an allergic immune response. ILC2s are activated by local immune mediators, typically alarmins, and are able to produce large amounts of signature cytokines within a short period of time (9). Thereby, ILC2s can initiate and amplify immune responses and are able to influence innate as well as adaptive immunity by both their secreted cytokines and through cell-cell interactions. Hence, ILC2s serve as an important link between innate and adaptive effector branches of type 2 immunity. Depending on the tissue they reside in, ILC2s exhibit slightly diverse profiles shaped by their microenvironment (10). However, their specific characteristics such as their ability to produce type 2 signature cytokines in a stark and fast fashion remains unchanged. Since their detailed description in 2010 (11–13), our knowledge about this fascinating immune population has steadily increased both in mouse models but also, through applied research, in humans. In this review we will provide a brief snapshot on our current knowledge of ILC2s in mouse and human allergic respiratory inflammation. Moreover, we will summarize experimental mouse models and discuss how recent reports led to an improved understanding of therapeutic strategies in human allergic respiratory diseases with the focus on asthma.

## Asthma – an Allergic Respiratory Disease

One of the most common human allergic diseases in the respiratory tract is asthma. Importantly, asthma is no longer considered to be one specific disease but more of an umbrella term for chronic inflammation of the lower airways with characteristics such as wheezing, bronchoconstriction and shortness of breath (4, 14). The heterogeneity of asthma is mirrored by different immune profiles. In general, asthma is subdivided into type 2 and non-type 2 with further separation of type 2 asthma in allergic or non-allergic asthma accompanied with eosinophilia (14, 15). Non-type 2 (low) asthma is defined as asthma without eosinophilia and with increased presence of neutrophils and/or IL-17 producing cells. These different asthma subtypes are termed endotypes for better classification (16). Interestingly, although there are cases of asthma onset in adulthood in a cohort of patients, most asthmatic individuals develop the disease during childhood (17). However, the exact mechanisms are still not completely understood. Different secondary diseases are correlated with asthma such as allergic rhinitis, chronic rhinosinusitis, and the development of nasal polyps. To provide optimal care to asthmatic patients of all different subgroups, a shift to a more personalized treatment approach is in the focus of discussion. In this context, mouse models of human allergic airway inflammation are an invaluable tool to understand underlying mechanisms of disease. Common allergens, including house dust mite (HDM) and papain can trigger respiratory allergic inflammation in humans as well as mice (18, 19), and mouse models can therefore be used to recapitulate these immune responses in an experimental system and thus aid in deciphering the underlying mechanism and processes of allergic respiratory diseases. To increase our knowledge of allergic lung inflammation, different experimental mouse models can be applied. Allergens such as ovalbumin (OVA), HDM, papain, fungal extracts, ILC2-eliciting cytokines and combinations thereof are used to induce and mimic allergic inflammation in the respiratory tract. The importance of ILC2s in the onset of allergic airway inflammation is highlighted by their detection in these experiments and thoroughly stratified in Table 1.

**Table 1:**
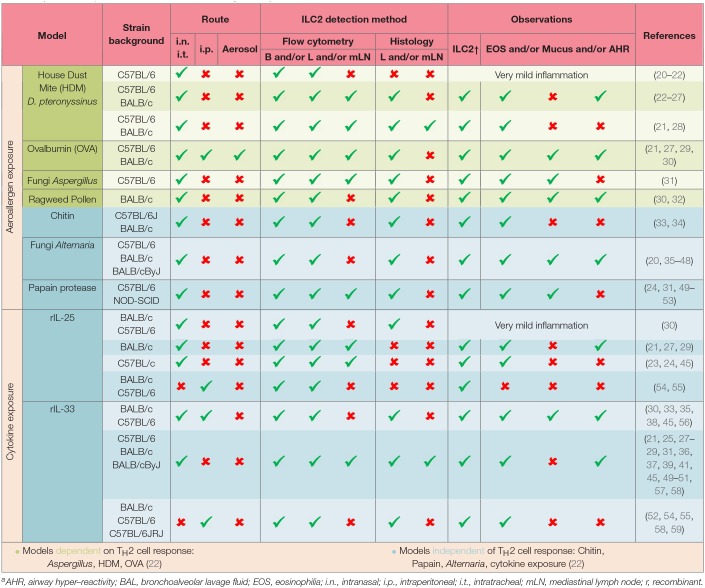
ILC2s in experimental mouse models of allergic airway inflammation^a^.

a *AHR, airway hyper–reactivity; BAL, bronchoalveolar lavage fluid; EOS, eosinophilia; i.n., intranasal; i.p., intraperitoneal; i.t., intratracheal; mLN, mediastinal lymph node; r, recombinant*.

## ILC2s in Allergic Inflammation of the Respiratory Tract

Allergic respiratory diseases are characterized in general by a dysregulated immune response targeting a harmless and non-pathogenic allergen. Several immune populations participate in an allergic respiratory disease including populations of the innate as well as the adaptive immune system. Different subgroups of T and B cells as well as eosinophils, basophils, NK cells and, last but not least, ILC2s are major players. Although a direct mechanistical link of ILC2s to asthma pathogenesis still needs to be established in humans, an increasing body of evidence supports an association of ILC2s with disease in asthmatic patients. These include GWAS studies revealing several genes essential in ILC2 biology as susceptibility markers for human asthma such as the ILC2-activating alarmin IL-33 and its cognate receptor ST2 (IL1RL1) as well as the transcription factors GATA3 and RORα (57, 60, 61).

ILC2s are the predominant ILC population in the lung, in contrast to other mucosal surfaces such as small intestine and colon. However, the reason for this skewed distribution is still not clear. ILC2s are activated mainly by the cytokines interleukin (IL)-25, IL-33, and thymic stromal lymphopoietin (TSLP) in the lungs, however, ILC2s accumulate in tissues independent of these signals and pulmonary ILC2s can also be detected in triple knockout mice deficient in the TSLP receptor, ST2, and IL-25 (10, 62). However, ILC2s show reduced IL-5 reporter expression in lung, fat, and gut tissue, but not in the skin indicating that TSLP, ST2, and IL-25 signaling is indeed needed for the functional maintenance of ILC2s in the local microenvironment of the lung (10). In addition to IL-5, ILC2s commonly secrete IL-4, IL-9, IL-13, and amphiregulin. IL-4 triggers the differentiation of T_H_2 cells and induces class switching of B cells to IgE. IL-5 activates B cells and plays a role in eosinophil homeostasis (63). IL-9 is needed by ILC2s for their survival and maintenance (64, 65), and IL-13 induces goblet cell hyperplasia and mucus secretion but can also act on alveolar macrophages (13, 66) and initiate the migration of dendritic cells to the mediastinal lymph nodes (49). Moreover, ILC2s are able to serve as antigen presenting cells for T cells by expressing MHCII (67), although this appears to be less pronounced in the lungs compared to the gut (67). Additional functional characteristics of pulmonary ILC2s include their expression of *Il5* as well as ST2 (IL-33R) at steady state in contrast to intestinal ILC2s which express both IL-5 and IL-13 mRNA and mainly the IL-25 receptor chain (IL-25RB) further demonstrating that ILC2s are imprinted by their microenvironment (10, 63).

**Figure 2:**
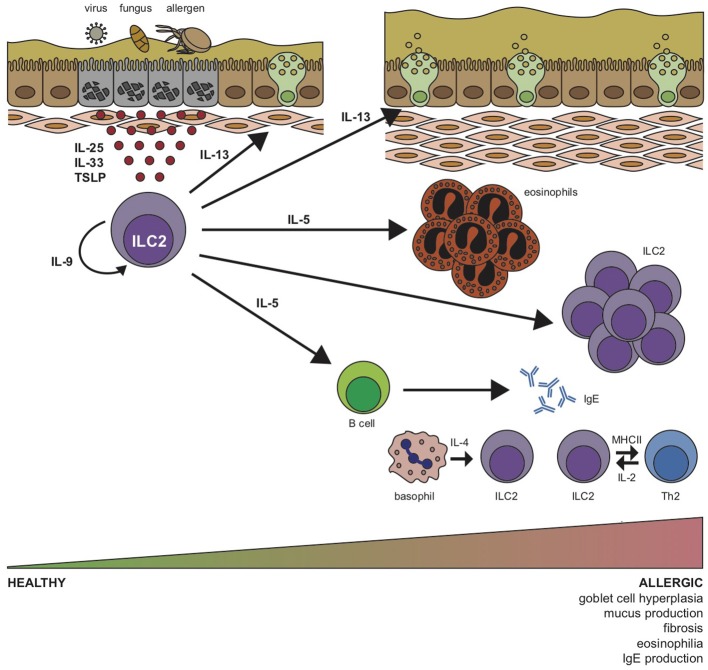
Group 2 innate lymphoid cell (ILC2) stimulation by airway pathogens drives allergic respiratory inflammation. In response to allergens, fungi or viruses, lung epithelial cells release alarmins IL-25, IL-33, and thymic stromal lymphopoietin (TSLP) that induce expansion and cytokine production by ILC2s. Activated ILC2s initiate an innate type 2 inflammatory response through production of IL-5, which induces eosinophilia, and IL-13, which promotes airway hyper-reactivity, goblet cell hyperplasia, mucus production and fibrosis. B cells and basophils are stimulated to release IgE and IL-4, respectively, and ILC2-derived IL-9 acts in an autocrine manner to prolong survival of ILC2s in the lung. Expression of MHCII enables ILC2 interaction with T_H_2 cells, which subsequently promote ILC2 function via T cell-derived IL-2.

Furthermore, ILC2s get support from basophil-derived IL-4 (68), and T cell-derived IL-2 (56), thus, establishing a quick but robust allergic reaction (Figure 2). In addition to the typical type 2 cytokines, IL-17 secreting ILC2s have been described, a cytokine known to be regulated by RORγt that has been correlated to severe asthma phenotypes (69). However, while one group reported IL-17 expression by KLRG1^hi^ST2^−^ inflammatory ILC2s (iILC2s) in the lungs, which correlated with their expression of RORγt (70) a more recent report showed increased IL-17^+^ST2^+^ ILC2s (ILC2_17_s) upon IL-33 or allergen challenge, independently of RORγt expression (52, 70). Finally, in line with their immunomodulatory potential, ILC2s can also acquire a regulatory phenotype and memory-like properties upon IL-33 and IL-2 stimulation *in vivo*, decreasing the expression of their pro-inflammatory repertoire as well as eosinophilic recruitment and accumulation (71). Further studies on this subset could certainly open up new venues in allergic disease therapies. Overall, ILC2s are key in the initiation, amplification, and modulation of type 2 immune responses in the respiratory tract by exhibiting these fundamental characteristics.

Due to their scarcity and phenotypical heterogeneity in mice and humans, the detection of ILC2s by flow cytometry requires some considerations. ILC2s are characterized by the absence of known lineage markers and antigen receptors as well as the expression of surface markers CD45, CD25 (IL-2Rα), CD127 (IL-7Rα), and ST2 (IL-33R) in human asthma as well as in rodent models of respiratory allergic inflammation. Interestingly, CD127 low/negative ILC2s have also been reported in asthmatic patients as well as in an allergic experimental mouse model (72, 73). Of note, iILC2s show reduced CD127 expression upon systemic challenge with IL-25 (70). Whether these ILC2s have just downregulated the expression or endocytosed CD127 due to strong activity still needs to be investigated. In addition, mouse ILC2s are further identified by the surface expression of Thy1, a marker that is not present on human ILC2s. Another difference is the expression of CD161 by human ILC2s. The functions of these two glycoproteins are still not completely understood. Both mouse and human ILC2s can express the prostaglandin receptor family member CRTH2. CRTH2 is important in the migration of ILC2s and thereby expressed to a different extent depending on location (74). However, the detection of mouse CRTH2 is limited by the availability of a specific detection antibody (74, 75). Our current strategies to detect ILC2s are largely based on surface receptors, which are actively used in an immune response by the cell to sense its environment and act accordingly. In addition, surface receptors and Gata3 are often used in combination to detect human and murine ILC2s (76, 77). The caveat with Gata3 is that ILC2s in the lungs are able to downregulate Gata3 expression under specific conditions such as viral (78), or helminth infections (70), however, under steady state conditions Gata3 is a reliable marker for pulmonary ILC2s.

Interestingly, ILC2s are not a homogenous population and several different subtypes of ILC2s have been reported at mucosal surfaces (79), including the lungs (31, 70). Memory ILC2s can be elicited upon repeated challenge of mice with IL-33, or when using papain as an allergen (31), thereby intensifying the immune response. Thus, ILC2s are specifically shaped by their local microenvironment and are reported to exhibit a sedentary lifestyle (45, 80). However, a subgroup of murine ILC2s, intestinally-derived inflammatory ILC2s (iILC2s) are able to traffic through the lymphatics and blood toward the lung to aid in the local response to helminth infection or systemic challenge with IL-25 (81). Moreover, the identification of circulating multipotent ILC progenitors in peripheral blood in humans indicates that ILC2 progenitors may be able to traffic to local pools of tissue-resident cells and replenish them (82). Since iILC2s are able to further differentiate into natural ILC2s (nILCs), the ability to traffic might be limited to specific circumstances and confined to not yet fully matured or differentiated ILC2 populations. However, additional studies are needed to pinpoint the potential of ILC2s to migrate within the body. nILC2s, which are present at steady state in the lungs and characterized by their ST2 expression, are typically increased upon stimulation with IL-33 (70). Both populations, iILC2s and memory ILC2s are characterized by elevated expression of the IL-25 receptor in the lungs (31, 70), however, it is still not completely understood how similar the biology and function of both populations is. So far, IL-25 receptor-expressing ILC2s have only been described in the human skin (83), and it still needs to be determined whether IL-25 receptor expression is present and regulated in human ILC2s during respiratory inflammation. KLRG1 is another commonly used marker to identify mature ILC2s in both human and mouse lungs. However, its expression is greatly influenced by androgens with ILC2s from male mice exhibiting higher expression of KLRG1 while female lungs harbor significantly higher numbers of KLRG1^−^ ILC2s promoting lung inflammation (59, 84). The ligand for KLRG1 is E-cadherin and ILC2s isolated from human skin were shown to be restrained by this interaction (83). Testosterone can additionally regulate ILC2s by controlling their response to IL-2 as well as by the restraint of IL-5 and IL-13 production and thus resulting in decreased pulmonary pathology upon *Alternaria* challenge (42). In the same study, elevated levels of ILC2s in peripheral blood of female asthma patients in comparison to male patients have been reported which is especially interesting in the light of the increased prevalence of asthma in women. The exact role of androgens such as testosterone in asthma still needs further investigation since testosterone is able to induce IL-33 mRNA in mast cells (85), however, lower levels of IL-33 and TSLP have been detected in the bronchoalveolar lavage of *Alternaria* challenged mice.

Insights into the function of ILC2s in allergic inflammation has mainly been generated using experimental mouse models. However, a substantial amount of reports provide evidence that ILC2s are also key in human allergic respiratory inflammation. Of note, the first reports on human ILC2s provided a detailed description of ILC2s in polyp tissue of chronic rhinosinusitis patients (75, 77). Moreover, increased levels and activity of ILC2s have also been reported in asthmatic patients. ILC2s could be detected in bronchoalveolar lavage, lung tissue, sputum and blood of patients with respiratory inflammation. Although the gating strategies of ILC2s slightly vary between the distinct reports, all studies demonstrate expression of CD127 in combination with CRTH2 and/or CD44 and ST2 on ILC2s. A positive correlation of eosinophilia and ILC2s levels has been further reported in human patients similar to the observation in mouse respiratory inflammation (86, 87). Recent work opened the discussion of functional redundancy of T_H_2 cells and ILC2s in humans (88). However, even if this is the case, pulmonary ILC2s have a critical function in the development of allergic diseases being innately committed to type 2 immunity and strong and immediate amplifiers of initial responses.

## Obesity-Associated Asthma

The prevalence of both obesity and asthma has increased drastically in recent years. Although asthma in obese patients is characterized mainly as non-allergic with an increase in neutrophils, eosinophils have been reported to be present in elevated numbers in the lung tissue of obese asthma patients as well (89). Of note, in addition to their mucosal location, ILC2s were originally identified as fat-associated lymphoid cluster (FALC) Lineage^−^ckit^+^Sca-1^+^ cells in the mesentery (12). Here, adipocytes and endothelial cells within the adipose tissue are sources of ILC2-activating IL-25 and IL-33 (90, 91). ILC2s are able to maintain the metabolic status of healthy adipose tissue by secreting IL-5 for eosinophil homeostasis, IL-13 to trigger alternative macrophage differentiation and methionine-enkephalin which directly acts on adipocytes and induces beiging of fat (92). However, in obesity ILC2s are decreased in adipose tissue and in ILC2-deficient mice, a high-fat diet accelerates obesity and insulin resistance indicating that ILC2s in adipose tissue are important for homeostasis. It thus seems contrary at first to link obesity and asthma. However, although obese mice have lower ILC2s and eosinophils in their adipose tissue, the levels of these populations are increased in the lungs in obese mice at steady state and upon allergen challenge such as with HDM. Not only ILC2s but also ILC3s are increased in the lungs of obese mice which can be enhanced by ozone triggered IL-33 or Nlrp3 inflammasome induced IL-1β, respectively (93, 94). It has been suggested that ILC2s and eosinophils might migrate from adipose tissue into lung tissue during obesity and thereby influence pulmonary immunity and may trigger asthma. This represents an interesting potential mechanism but further research will be needed to fully support this idea.

## Biomarkers and Assessment of Severity of Allergic Respiratory Diseases

Interestingly, in humans, an allergic response is provoked in the skin as a first assessment of an allergic response in general but also as a first evaluation of asthma. Different tests can be used depending on the way of application using subcutaneous injections or exposure to allergen by topical application with the most common test in clinical practice still being the prick test. It still needs to be determined to what extent and how ILC2s directly contribute to the assessment of allergy via the prick test. Upon external stimuli or cellular damage, IL-33 is released from cells and engages ST2 on ILC2s but also on T_H_2 cells, eosinophils, mast cells, and basophils, contributing to cutaneous allergic inflammation with increased levels of local and peripheral blood ILC2s, eosinophils, IgE, and histamine (95). IL-33 can be released locally and systemically after mechanical skin injury (i.e., scratching), promoting IgE-mediated degranulation (96). Of note, ILC2s present in the skin can control mast cell (MC) activity by direct interaction (97). Conversely, MC are an important source of IL-33 *in vivo*, contributing to ILC2 activation and type 2 immune response in disease models of multiple sclerosis (85), and helminth infection (98), a MC-ILC2 crosstalk also occurs in the lung (99). It is not yet fully understood how cutaneous antigen exposure could activate ILC2s but it is conceivable that dysregulation between MC and ILC2s could exacerbate the immune response during allergic airway inflammation and anaphylactic reaction. Moreover, the evaluation of the eosinophil count, as well as the level of IgE, including allergen-specific IgE in blood, or less common in sputum, is used to assess the grade of the allergic response. Type 2 signature cytokines (IL-4, IL-5, and IL-13) are used in addition as biomarkers. However, to assess the severity of asthma, lung function tests are routinely carried out in humans. Sequential examinations are performed on the patient such as allergy tests to pinpoint the responsible allergen(s), bronchial provocation or exhaled nitric oxide tests; before formulating any therapeutic recommendation (100). Since asthma and allergic inflammation of the respiratory tract can have multiple underlying causes, the aim is to personalize the treatment as much as possible depending on the results of the examinations. Thus, achieving control is the main objective currently proposed in asthma management where pharmacological and non-pharmacological treatment is adjusted in a continuous cycle that involves assessment, treatment and review. In mouse models, experimentally-induced airway hyper-reactivity is usually analyzed upon challenge with increasing doses of inhaled methacholine. The concept of using ILC2 prevalence as a biomarker for diagnostic purposes in lung disease is appealing. Screening of ILC2s as an early hierarchical population might already indicate asthma susceptibility before the start of symptoms or pathology in the lungs. However, due to their high phenotypic diversity, ILC2 characterization in different asthmatic subgroups and their comparison is necessary, and even then, ILC2 level and functionality should be carefully evaluated for each subgroup. Nevertheless, biomarkers and the assessment of allergic respiratory diseases show overall important similarities between rodent models of disease and clinical practice.

## Experimental and Therapeutic Strategies to Ameliorate Respiratory Inflammation

### Corticosteroids

In humans, as a first and often immediate treatment inhaled corticosteroids are commonly used in both allergic and non-allergic respiratory inflammation. Corticosteroids reduce the general inflammation of the lung and provide relief of symptoms for the patient (101), but can also cause adverse side effects especially when given systemically in high doses and during long-term treatment. Importantly, corticosteroids dampen the activity of both mouse and human ILC2s (102). However, under specific conditions, corticosteroids are less able to act on ILC2 activity. This has been reported to be the case in situations of enhanced STAT5 activation upon TSLP stimulation. This increased activation of STAT5 by TSLP has been identified as a regulatory mechanism in mice (102), was further confirmed in human ILC2s (103) and helps to explain why not all asthma patients respond to corticosteroid therapy. Corticosteroid resistance can also occur in neutrophilic asthma. IL-17 contributes to neutrophil accumulation in the lungs but also increases the expression of the glucocorticoid receptor beta (GRβ) (104). GRβ inhibits the activity of GRα by direct competition for glucocorticoids. Increased expression of GRβ has been reported on cell populations in glucocorticoid-resistant patients (105, 106), and is discussed to contribute to steroid resistance in neutrophilic asthma (104). IL-17 can also be derived from pulmonary iILC2s (70) as well as ILC2_17_s, which were described to be the main source of IL-17 in the lung after IL-33-induced lung inflammation (52). However, if and which role ILC2s may have in neutrophilic asthma still needs to be investigated.

### Adrenergic Agonists (β_2_-Agonists)

Like corticosteroids, β_2_-agonists that act on β_2_-adrenergic receptors are frequently used to treat asthmatic patients. A recent report showed that both human and mouse ILC2s express the β2AR (β_2_-adrenergic receptor) for epinephrine and norepinephrine and that the use of an agonist during lung inflammation in mice impaired ILC2 proliferation, cytokine production and effector function (107). These findings highlight the importance of ILC2s in integrating neuroimmune signals. In humans, short acting β-agonists (SABA, e.g., Salbutamol) and long acting β-agonists (LABA, e.g., Salmeterol) are routinely used for asthma treatment. LABA is used in combination with inhaled corticosteroids whereas SABA is also approved as a monotherapy in mild asthma (108). Adverse effects of β_2_-agonists have been reported and the discussion was stirred up upon the report of detrimental effects upon excessive Fenoterol (SABA) treatment (long time and high dose) (109, 110). The underlying mechanisms of these severe consequences are only incompletely understood. However, the use of β_2_-agonists for asthmatic patients under treatment is generally regarded as safe and successful (101).

### Lipid Mediators: Leukotrienes & Prostaglandins

Bioactive lipid mediators are important regulators of ILC2s (111). Indeed, leukotrienes (LTs) including cysteinyl LTs (CysLTs) are generated by arachidonic acid metabolism and CysLTs have been linked to the initiation of asthma and bronchoconstriction since a long time (112). Leukotriene receptor antagonists such as Montelukast, a CysLT_1_ receptor antagonist, are commonly prescribed to improve asthma symptoms in humans (113). Both mouse and human ILC2s have been reported to express CysLT receptors and it was shown that CysLTs positively regulate ILC2 activation (47, 114–116).

In addition, Leukotriene B4 (LTB4) has also been linked to asthma. LTB4 is a neutrophil chemoattractant in pulmonary inflammation (117), and neutrophils can be increased during the exacerbation phase of asthma (118). However, sputum samples of asthmatic patients showed an increased level of LTB4 which strikingly did not correlate with neutrophil levels in the samples of the analyzed patients (119). The high affinity receptor for LTB4, LTB4R1, has been reported on mouse ILC2s but its presence and role in human ILC2s still needs to be elucidated. Interestingly, LTB4 also plays an important role in the development of insulin resistance in obese mice (120). However, how and if ILC2s are involved in this context still needs to be addressed.

Similar to LTs, prostaglandins are products of arachidonic acid metabolism. As mentioned previously, human and mouse ILC2s express the receptor for prostaglandin D2, CRTH2 (74, 75). CRTH2 plays a potent role in activation, migration and cytokine release of T_H_2 cells and eosinophils. Curiously, accumulation of pulmonary ILC2s is regulated via CRTH2 and differences in its expression on ILC2s have been reported in inflamed pulmonary tissue (121). Use of monoclonal antibodies against CRTH2 resulted in a reduction of CRTH2 expressing cells including ILC2s in mice (122). In addition to its role in migration of ILC2s, PGD_2_ has been reported to potentiate the action of ILC2s eliciting cytokines leading to an increase in effector cytokine expression. Consequently, small-molecules antagonists of the CRTH2 receptor, have been promising in human trials for asthma patients (123–126). One example is OC000459 which was reported to be a safe and effective alternative treatment of eosinophilic asthma improving lung function and asthma symptoms (127, 128).

### Signature Cytokines and IgE

Monoclonal antibodies are used to treat allergic respiratory diseases such as asthma in humans. These antibodies are directed against either type 2 signature cytokines IL-4, IL-5, and IL-13, their respective surface receptors or against IgE. Both T_H_2 cells and ILC2s are potential sources of type 2 signature cytokines. Moreover, ILC2s have been reported to enhance the adaptive immune response and thereby influence IgE production. The following monoclonal antibodies to treat asthma are currently used in clinical practice: Dupilumab (moderate to severe asthma and severe asthma) targets IL-4 and IL-13 by binding to the IL-4Rα subunit thereby acting as a blocking antibody for these signaling pathways (129, 130). Mepolizumab and Reslizumab (both eosinophilic asthma) target IL-5 directly and thereby neutralize this signature type 2 cytokine, reducing the rate of exacerbations (131, 132). Benralizumab (severe eosinophilic asthma) is directed against the IL-5Rα subunit (133). Lebrikizumab (severe asthma) and Tralokinumab (moderate to severe asthma) are directed against IL-13 (134, 135), and Omalizumab (severe allergic asthma in adults and children) targets and neutralizes IgE (136, 137). The ILC2-eliciting cytokine TSLP plays a critical role in human asthma and antibodies to neutralize TSLP (Tezepelumab) have been tested to treat allergen-induced asthma (138, 139). Overall, these antibodies block and thereby neutralize important immune mediators secreted by ILC2s as well as T_H_2 cells.

### Neuropeptides

ILC2s in mouse and human can sense and respond to neuropeptides such as neuromedin U (25, 140, 141). Although pulmonary ILC2s exhibit a more moderate response to neuromedin U when compared to intestinal ILC2s under the tested conditions (140), several different neuropeptides are present in the lungs (142), including vasoactive intestinal peptide (VIP). VIP can induce cytokine stimulation of intestinal but also pulmonary ILC2s (63). Inhalation of a VIP agonist (Ro 25-1553) resulted in a short but significant bronchodilatory effect (143), however the exact mechanism and a possible link to pulmonary ILC2s is unclear. In contrast to VIP, calcitonin gene-related peptide (CGRP) is elevated in some asthmatic patients (144), and neuroendocrine cells, which co-localize in the airways with ILC2s are an important source thereof (145). ILC2s respond to CGRP by secreting more IL-5, however, targeting CGRP and thereby ILC2s still needs to be evaluated in asthma patients. Targeting neuropeptides and their receptors may be a promising concept for future therapy but still requires further investigation.

### Transcription Factors

GATA3 is the master transcription factor of ILC2s and critical in regulating asthmatic responses in patients with a predominant T_H_2 phenotype. Targeting GATA3 is complex due to its intranuclear location but would enable to already intervene at a very early stage in the disease formation process. Novel approaches to antagonize GATA3 using antisense molecules (DNAzymes) overcome this challenge by cleaving and inactivating GATA3 messenger RNA (mRNA). GATA3-specific DNAzyme SB010 has shown to significantly attenuate both early and late-phase asthmatic responses after allergen exposure in a phase IIa proof-of-concept trial (146).

## Concluding Remarks & Outlook

Since allergic respiratory diseases are rapidly increasing worldwide, there is a critical need to optimize current and develop novel therapeutic strategies. Reports in recent years have shown that ILC2s are important players in experimental mouse models of allergic airway inflammation and their role in asthmatic patients is just starting to unveil itself. Although neutralizing ILC2-elicited immune mediators or blocking respective signaling pathways are currently used to treat asthma, it is of great interest to develop alternative strategies to target not only the consequence but the cause of respiratory inflammation. This may be achieved by blocking ILC2s fairly early in disease and re-directing their activity. Moreover, the merge of immunology with other fields such as neurobiology opens new concepts and will reveal novel targets of translational interest. The unique microenvironment of the respiratory tract with its diversity of non-hematopoietic cells and their close proximity to ILC2s will as well unfold thrilling answers of ILC2 maintenance and activation in the future. We are excited to see further research on ILC2 biology in respiratory allergic inflammation which will surely provide essential knowledge to develop novel concepts and strategies for asthma treatment and improving overall respiratory health.

## Author Contributions

All authors listed have made a substantial, direct and intellectual contribution to the work, and approved it for publication.

### Conflict of Interest Statement

The authors declare that the research was conducted in the absence of any commercial or financial relationships that could be construed as a potential conflict of interest.
